# Real-World Overall Survival and Time to Next Treatment Among Medicare Beneficiaries with Chronic Lymphocytic Leukemia in the Frontline Setting

**DOI:** 10.3390/cancers18121902

**Published:** 2026-06-11

**Authors:** Scott F. Huntington, Justin T. Puckett, Beenish S. Manzoor, Nnadozie Emechebe, Sophia S. Li, Sachin Kamal-Bahl, Carolina Reyes, Holly Budlong, Jalpa A. Doshi

**Affiliations:** 1 School of Medicine, Yale University, 37 College St., Rm 141, New Haven, CT 06520-8073, USA; 2 COVIA Health Solutions, Lansdale, PA 19446, USA; 3 AbbVie Inc., North Chicago, IL 60064, USA; 4 Genentech Inc., South San Francisco, CA 94080, USA; 5 University of Pennsylvania, Philadelphia, PA 19104, USA

**Keywords:** chronic lymphocytic leukemia, venetoclax, Bruton tyrosine kinase inhibitors, frontline therapy, Medicare, real-world outcomes, survival

## Abstract

**Background/Objectives**: Covalent Bruton’s tyrosine kinase inhibitors (cBTKis) and the BCL-2 inhibitor venetoclax (VEN) have largely replaced traditional chemotherapy in clinical guidelines for frontline treatment of chronic lymphocytic leukemia (CLL). However, real-world evidence comparing these therapies on key outcomes remains limited. This study examined overall survival (OS) and time to next treatment (TTNT) among U.S. Medicare beneficiaries initiating frontline CLL therapy. **Methods**: In this retrospective study using 2016–2023 national 100% Medicare fee-for-service claims, we identified 10,949 patients aged ≥65 who initiated CLL treatment between June 2019 and December 2022. Patients received VEN-based regimens (n = 1503), cBTKi-based regimens (n = 5956), or other regimens (n = 3490). **Results**: Kaplan-Meier analyses showed superior OS for VEN-based regimens compared with cBTKi-based regimens and other regimens; three-year survival was 77% for VEN, 67% for cBTKis, and 62% for other regimens. In Cox regression models, cBTKis (hazard ratio [HR] 1.48, 95% CI 1.31–1.67) and other therapies (HR 1.66, 95% CI 1.47–1.89) were associated with worse OS relative to VEN. TTNT results also favored VEN; the percentage of patients with no evidence of second-line treatment at 3-years higher for VEN-based regimens (86%) compared to cBTKi-based regimens (69%) and other regimens (52%), despite similar follow-up times (median [months] VEN = 26.6, cBTKis = 26.2, Other = 21.8). cBTKis (HR 2.69, 95% CI 2.23–3.26) and other regimens (HR 6.47, 95% CI 5.32–7.86) were associated with a greater hazard for subsequent treatment relative to VEN. **Conclusions**: Our study demonstrates better survival and reduced need for subsequent treatment with VEN in patients with CLL treated in the frontline setting.

## Simple Summary

Treatment for chronic lymphocytic leukemia has changed greatly in recent years, with newer oral medicines replacing older chemotherapy approaches for many patients. However, less is known about how these treatments perform in everyday practice, especially in older adults. In this study, we looked at Medicare patients aged 65 years and older who started their first treatment for chronic lymphocytic leukemia in the United States. We compared patients who received venetoclax-based treatment, Bruton tyrosine kinase inhibitor-based treatment, and other types of therapy. Patients who received venetoclax-based treatment lived longer and were less likely to need another treatment later than those in the other groups. These results provide real-world information on how these treatment options work in older patients and may help doctors and researchers make more informed decisions about treatment choice and future studies.

## 1. Introduction

Chronic lymphocytic leukemia (CLL) is the most common adult leukemia in the United States, with a median age at diagnosis of approximately 70 years [1,2]. The disease predominantly affects older adults, a population often characterized by a higher comorbidity burden, polypharmacy, and increased vulnerability to treatment-related toxicities [3,4]. These factors can complicate therapeutic decision-making and influence both the tolerability and effectiveness of treatment [5].

Over the past decade, treatment strategies for older adults with frontline CLL have shifted dramatically away from traditional chemotherapy/chemoimmunotherapy (CT/CIT), such as chlorambucil-based combinations, bendamustine plus rituximab, and fludarabine-based regimens, toward innovative targeted therapies [6]. This shift in the treatment paradigm began with the March 2016 FDA approval of the covalent Bruton’s tyrosine kinase inhibitor (cBTKi) ibrutinib for first-line CLL treatment. Bruton’s tyrosine kinase (BTK) is a key signaling protein in the B-cell receptor pathway that supports survival and proliferation of malignant B cells in CLL, and cBTK inhibitors target this pathway to disrupt downstream signaling. Second-generation cBTKis, such as acalabrutinib (approved November 2019) and zanubrutinib (approved January 2023), followed in subsequent years [7]. These newer agents are more selective for BTK and have demonstrated improved tolerability compared with ibrutinib [7]. In addition to these cBTKi-based regimens, the May 2019 approval of the B-cell lymphoma 2 inhibitor (BCL-2i) venetoclax (VEN) in combination with obinutuzumab offered the first non-cytotoxic fixed duration treatment (FTD) option for older adults in frontline CLL [8]. BCL-2 is an anti-apoptotic protein overexpressed in CLL cells, and VEN promotes apoptosis by selectively inhibiting the anti-apoptotic BCL-2 protein overexpressed in CLL cells. Randomized clinical trials have demonstrated these agents’ ability to improve progression-free survival (PFS) compared to CT/CIT with manageable toxicity profiles [9,10,11]. As a result, major clinical guidelines now recommend VEN-based regimens and cBTKi-based regimens as preferred frontline options for most patients [12].

However, the benefits observed in clinical trials may not fully translate to routine clinical practice, particularly in older patients with multiple comorbidities and more heterogeneous health profiles [13,14,15,16]. Real-world evidence (RWE) is essential for evaluating the effectiveness and durability of these agents in diverse populations beyond clinical trials. Such evidence is especially relevant for older adults, who may differ substantially from trial participants in functional status, socioeconomic factors, and treatment adherence [17,18,19,20,21,22]. Understanding how novel therapies perform in the real-world setting may help guide evidence-based treatment selection and optimize patient outcomes. Preliminary findings from this Medicare cohort were previously presented in abstract form, although comprehensive comparative evidence remains limited [23]. This study sought to address this gap in the evidence base by examining overall survival (OS) and time to next treatment (TTNT) in a national sample of fee-for-service U.S. Medicare beneficiaries initiating VEN-based regimens, cBTKi-based regimens, or other regimens for CLL in the frontline setting with expanded multivariable and sensitivity analyses beyond those included in the prior abstract.

## 2. Materials and Methods

### 2.1. Study Design and Data Source

We conducted a retrospective cohort study using 2016–2023 100% Chronic Conditions Warehouse (CCW) Medicare claims data available from the Centers for Medicare & Medicaid Services (CMS). The data include Medicare Part A and Part B medical claims for inpatient and outpatient services, as well as Part D prescription drug event files for outpatient prescription fills for all fee-for-service Medicare beneficiaries. The Medicare claims files are linked to beneficiary summary files that contain patient demographic and enrollment details, as well as the date of death information.

### 2.2. Sample Selection

All Medicare beneficiaries aged ≥65 years initiating treatment for frontline CLL between 1 June 2019 and 31 December 2022 were included in the study. The index date was the date of the first prescription fill or infusion; all therapies received within 30 days of the index date were classified as the patient’s frontline treatment regimen [17]. Additional details regarding the identification of frontline CLL patients and the methods used to define lines of therapy are provided in the Supplementary Methods and Tables S1 and S2.

### 2.3. CLL Treatment Groups

CLL treatment regimens were classified based on clinical guidelines: [12] venetoclax- (VEN-) based, cBTKi-based (ibrutinib-, acalabrutinib-, and zanubrutinib-based regimens), and other regimens (chemotherapy/chemoimmunotherapy [CT/CIT] and anti-CD20 monotherapy). Patients receiving VEN with a cBTKi (n = 24) were included in the VEN-based group, given that these are fixed-duration treatment regimens.

### 2.4. Outcomes

The primary outcomes of interest were overall survival (OS) and time-to-next-treatment (TTNT), which was evaluated as a pragmatic real-world proxy for progression-free survival in the absence of direct progression data in the Medicare claims database. OS was defined as the time from each patient’s index date to the date of death as recorded in the Medicare Beneficiary Summary File. TTNT was defined as the time from initiation of the first-line (1L) regimen to the initiation of second-line (2L) treatment. Adding new therapy after 30 days indicated a new line, with the exception of anti-CD20 agents that could be added within 90 days. Discontinuation was defined as a 90-day gap in therapy. Further methodological details, including criteria for intra-class switching, are included in the Supplementary Methods.

### 2.5. Analysis

Landmark and median estimates for OS and TTNT by treatment group were calculated using Kaplan-Meier methodology. Cox regression models, adjusted for sociodemographic and clinical factors, were used to estimate hazard ratios and 95% confidence intervals. Details on censoring are provided in the Supplementary Methods.

Two sensitivity analyses were performed to evaluate the robustness of the primary findings under alternative clinically relevant assumptions. In the first sensitivity analysis, we restricted the VEN-based regimen group to those receiving venetoclax plus obinutuzumab (V + O) consistent with the FDA label in the frontline setting, to evaluate whether findings were consistent with the most commonly used fixed-duration regimen. In the second sensitivity analysis, intra-class switching of cBTKis (e.g., a switch from ibrutinib to acalabrutinib) did not trigger a new line of therapy; this sensitivity analysis was necessary to ensure our TTNT measure could serve as a proxy for progression-free survival, given switches within the cBTKi class may be due to tolerability issues rather than disease progression.

All analyses were conducted in SAS Enterprise, Version 9.4. Per CMS cell suppression and patient privacy policy, no results for <11 patients could be reported. The study was deemed exempt from review by the Pearl Institutional Review Board.

## 3. Results

**Figure 1:**
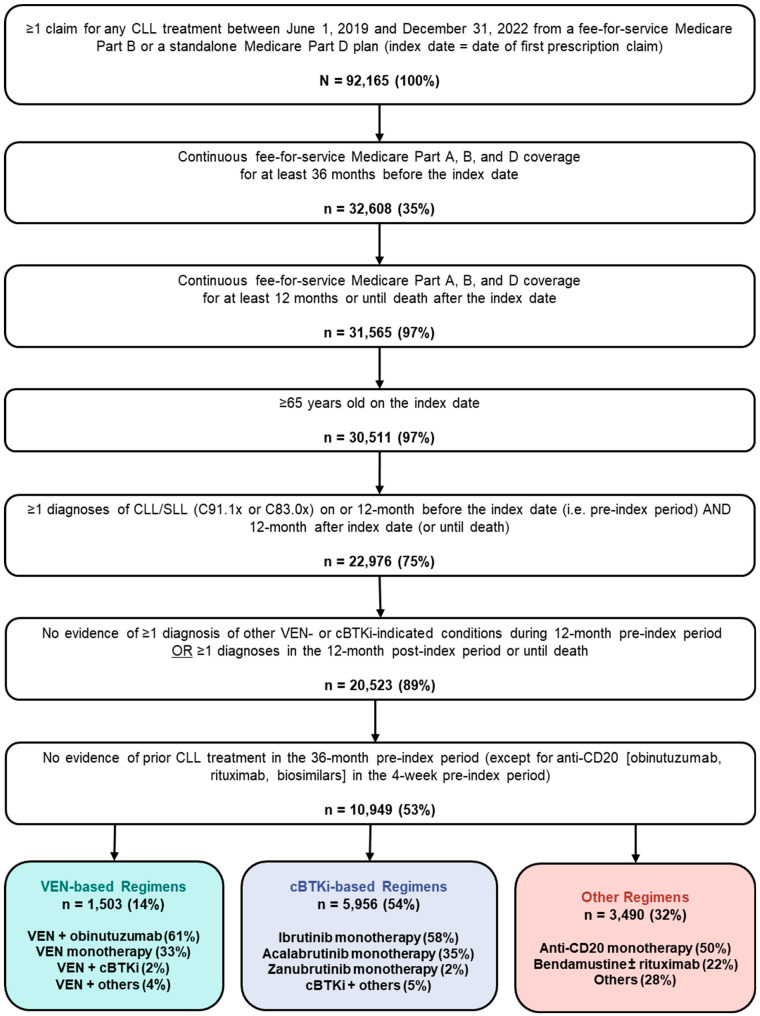
Sample Selection.

The final sample included 10,949 Medicare beneficiaries receiving frontline therapy for CLL (Figure 1). Among these, 13.7% received VEN (n = 1503), 54.5% received cBTKis (n = 5956), and 31.8% received other CLL therapies. Among VEN patients, most received V + O (60.6%) or VEN monotherapy (33.4%); a small number of patients received VEN in combination with a cBTKi (1.6%) or other treatments (4.4%). Among cBTKi patients, patients received ibrutinib monotherapy (58.0%), acalabrutinib monotherapy (34.9%), zanubrutinib monotherapy (2.3%), or a cBTKi in combination with other treatments (4.8%). Among patients receiving other CLL treatments, half (49.7%) received anti-CD20 monotherapy, while the rest received traditional CT/CIT. Across treatment groups, patients were predominantly White (92.1% [VEN], 91.7% [cBTKi], 92.4% Other), male (64.1% [VEN], 56.5% [cBTKi], 55.3% [Other]), and resided in urban areas (79.6% [VEN], 80.0% [cBTKi], 81.1% [Other], Table 1). The mean (SD) age for each group was 76.2 (5.8) years for VEN, 78.3 (6.6) years for cBTKis, and 78.3 (6.5) years for other. The comorbidity burden was considerable, with over half of patients having evidence of five or more Elixhauser comorbidities in the 12-month pre-index period: 54.7% (VEN), 54.1% (cBTKi), and 66.6% (Other). Median (IQR) follow-up after treatment initiation was 26.6 (16.8, 36.7) months for the VEN cohort, 26.2 (15.3, 36.7) months for the cBTKi cohort, and 21.8 (13.7, 36.0) months for patients receiving other therapies. During follow-up, the mean (SD) number of cBTKi or VEN prescription claims (not including claims for other agents in the regimen) was 15.3 (12.6) for cBTKis and 11.2 (7.8) for VEN.

### 3.1. Overall Survival

**Figure 2:**
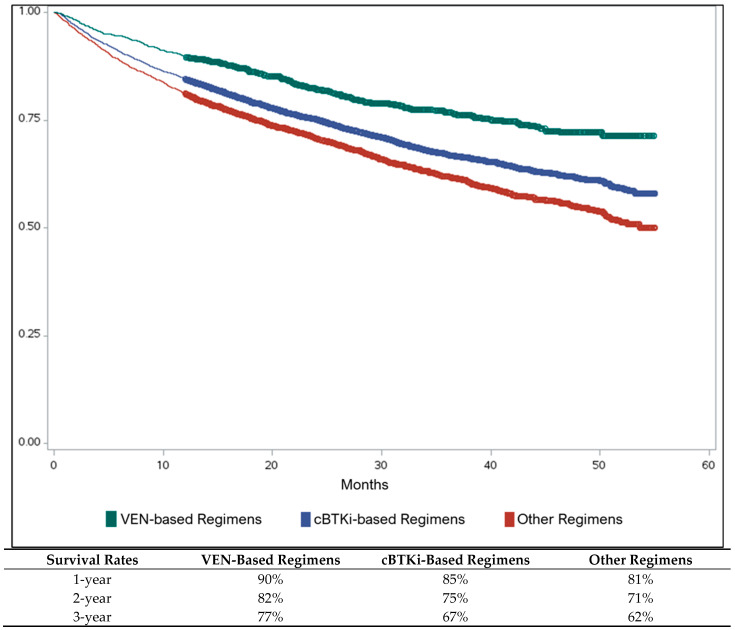
Kaplan-Meier Curve of Overall Survival among Medicare Beneficiaries with Frontline CLL.

Kaplan-Meier analyses demonstrated longer OS for patients treated with VEN compared with cBTKis and other CLL therapies (Figure 2). The 1-year and 3-year OS rates were 90% and 77% for VEN, 85% and 67% for cBTKis, and 81% and 62% for other therapies, respectively. Cox proportional hazards models confirmed that, relative to VEN, cBTKi use was associated with a 48% increased risk of death (hazard ratio [HR] 1.48, 95% CI 1.31–1.67, *p* < 0.001), while other CLL therapies were associated with a 66% increased risk (HR 1.66, 95% CI 1.47–1.89, *p* < 0.001, Table 2). Across the overall cohort, OS decreased with increasing age: compared with patients aged 65–69 years, those aged 75–79 years had worse survival (HR 1.56, 95% CI 1.31–1.85, *p* < 0.001), as did patients aged ≥80 years (HR 2.67, 95% CI 2.25–3.15, *p* < 0.001). Male patients, rural patients, low-income patients, patients with a greater comorbidity burden, and patients with CLL-related hospitalization in the pre-index period had worse survival; no statistically significant differences in OS were observed by race/ethnicity.

### 3.2. Time to Next Treatment

**Figure 3:**
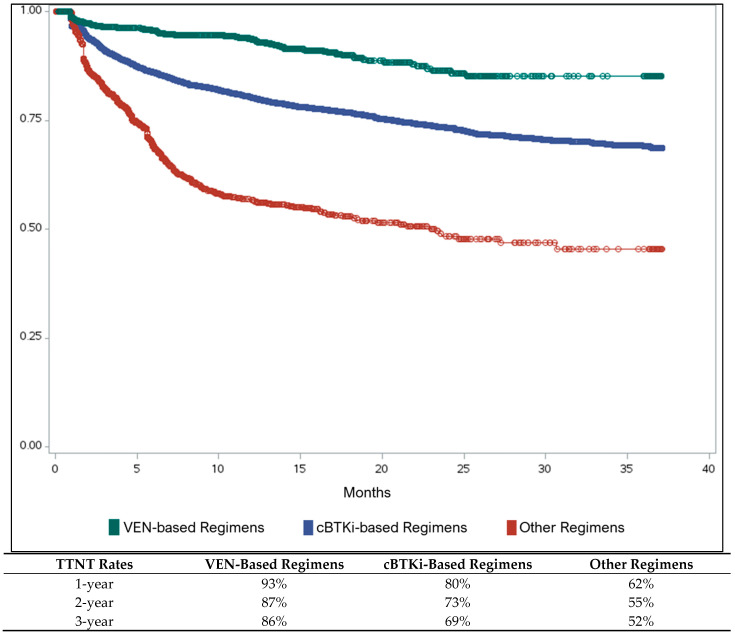
Kaplan-Meier Curve of Time-to-Next-Treatment among Medicare Beneficiaries with Frontline CLL.

KM analyses of time to next treatment (TTNT) similarly showed superior outcomes for VEN relative to cBTKis and other therapies (Figure 3). At 1 year and 3 years, the proportions of patients without evidence of second-line therapy were 93% and 86% for VEN, 80% and 69% for cBTKis, and 62% and 52% for other therapies. In Cox regression models, cBTKi use was associated with shorter TTNT compared with VEN (HR 2.69, 95% CI 2.23–3.26, *p* < 0.001), and other CLL therapies were associated with the greatest risk of needing subsequent therapy (HR 6.47, 95% CI 5.32–7.86, *p* < 0.001, Table 3). TTNT did not differ significantly by age or race/ethnicity.

### 3.3. Sensitivity Analyses

In the first sensitivity analysis, restricting the VEN cohort to patients receiving venetoclax plus obinutuzumab (V + O; n = 910, 61% of the VEN group), TTNT was considerably superior for V + O compared to cBTKis (HR: 3.55, 95% CI 2.69–4.68, *p* < 0.001) and other CLL treatments (HR: 8.56, 95% CI 6.47–11.31, *p* < 0.001). Results were also slightly improved in V + O patients with respect to OS, showing superior OS for V + O compared to cBTKis (HR: 1.61, 95% CI 1.37–1.89, *p* < 0.001) and other CLL treatments (HR: 1.81, 95% CI 1.53–2.13, *p* < 0.001; see Supplementary Figures S1 and S2 and Tables S3 and S4).

In the second sensitivity analysis wherein intra-class switching of cBTKis did not trigger a new line of therapy, TTNT increased for the cBTKi group from the primary analysis (84% and 80% for cBTKis at 1 year and 3 years), but descriptive and regression results confirmed that VEN was associated with better TTNT than cBTKis (HR 2.04, 95% CI 1.68–2.48, *p* < 0.001) and other CLL therapies (HR 6.52, 95% CI 5.35–7.95, *p* < 0.001) (Supplementary Figure S3 and Table S5).

## 4. Discussion

In this national cohort of older U.S. adults treated for CLL in the frontline setting, we found that treatment with VEN-based regimens was associated with significantly longer OS and TTNT compared with cBTKi-based regimens and other therapies. Our results are consistent with recently reported Phase 3 trials, such as CLL17, which demonstrated that fixed-duration treatment with VEN-based regimens is non-inferior to continuous treatment with ibrutinib in frontline CLL [24]. Importantly, our study expands upon these findings by demonstrating superior OS with VEN-based regimens compared to cBTKi-based regimens in—real-world clinical practice—providing important evidence that VEN-based strategies, including fixed-duration V + O, yield favorable outcomes for older adults initiating frontline therapy. Notably, the advantage for VEN persisted in sensitivity analyses restricted to VEN plus obinutuzumab and accounting for intra-class cBTKi switching, supporting the robustness of the observed findings. Our finding of improved TTNT is consistent with two smaller real-world studies comparing venetoclax-obinutuzumab versus cBTKi [8,25], with our analysis having the added strength of a considerably larger cohort with longer follow-up that enabled survival analysis in addition to TTNT.

Our real-world findings are broadly consistent with pivotal frontline CLL trials, although direct comparisons should be interpreted cautiously given differences in study design, patient populations, and endpoint definitions. In CLL14, fixed-duration venetoclax plus obinutuzumab demonstrated durable disease control, with a 3-year PFS rate of approximately 82% and a 6-year OS rate of approximately 79% in patients with previously untreated CLL and coexisting conditions [11,26]. In our real-world Medicare cohort, VEN-based regimens were associated with a 3-year TTNT rate of 86% and a 3-year OS rate of 77%, supporting the effectiveness of VEN-based therapy in routine clinical practice. In sensitivity analyses restricted to patients receiving fixed-duration V + O, outcomes remained favorable, with 3-year TTNT and OS rates of 88% and 79%, respectively, further supporting the durability and effectiveness of frontline V + O in older adults treated in routine clinical practice. For cBTKi-based therapy, RESONATE-2 reported a 5-year PFS rate of 70% and a 5-year OS rate of 83% with ibrutinib [9,27], while ELEVATE-TN reported a 2-year PFS rate of 93% for acalabrutinib plus obinutuzumab and later 6-year PFS and OS rates of approximately 78% and 84%, respectively [10,28]. Compared with these trial benchmarks, our real-world cBTKi cohort had a lower 3-year TTNT rate of 69%, which may reflect greater clinical heterogeneity, such as older age, higher comorbidity burden, and treatment discontinuation for tolerability or other non-progression reasons. Notably, about one-third of patients in CLL14 and ELEVATE-TN were aged 75 years or older [10,26], and 71% of RESONATE-2 participants were aged 70 years or older [9], whereas our real-world Medicare FFS cohort was substantially older, with approximately 56% of Ven-based patients and 68% of cBTKi-treated patients aged 75 years or older. In contrast to historical trial data, such as CLL11, which reported a median PFS of 26.7 months and a 5-year OS rate of 66% with obinutuzumab plus chlorambucil [29,30], our broader “other regimens” cohort had a 3-year OS rate of 62% [10,27]. Although this comparison is only descriptive, it suggests that outcomes with other frontline regimens in routine practice may be less durable than those observed in trials. These comparisons should be interpreted cautiously, however, because TTNT is not directly equivalent to protocol-defined PFS, and the observed estimates may also reflect treatment tolerability, adherence, clinical decision-making, and the greater clinical complexity of older real-world Medicare populations.

Our findings have implications for both payers and patients. For payers, VEN-based therapies may offer improved cost-savings by offering a fixed-duration regimen that delays disease progression (including during a considerable off-treatment period) and reduces the need for subsequent treatments. Real-world analyses have demonstrated that patients treated with VEN have lower rates of initiating additional therapy after discontinuation compared to patients treated with cBTKis [17]. In addition, real-world evidence from the Medicare population shows that the fixed-duration nature of VEN contributes to substantial cost savings compared with continuous cBTKi therapy, with estimated monthly all-cause healthcare costs after completion of the fixed-duration treatment approximately $8000 lower for patients receiving venetoclax plus obinutuzumab relative to those treated with a cBTKi [28]. This prior work also found that monthly out-of-pocket (OOP) costs faced by the patient while on treatment may be lower for patients treated with VEN ($581/month) compared to cBTKis ($703/month) [28]. While the implementation of an annual OOP maximum under Medicare Part D beginning in 2025 will lower OOP costs for both VEN and cBTKi patients, previous work has shown that VEN patients will continue to have lower costs over time compared to cBTKi patients due to the fixed duration of VEN treatment [29]. Beyond economic considerations, patient preferences are central to treatment decision-making. A discrete-choice experiment by Ravelo et al. (2024) highlighted that CLL patients prioritize therapies offering a higher likelihood of two-year progression-free survival [30]. Taken together, these findings suggest that VEN-based regimens not only provide clinical benefits but also align with both payer and patient priorities, supporting their use as a frontline treatment option for older adults with CLL.

Importantly, our study also challenges a common misperception that older adults may have difficulties with regimens such as V + O that include an infusion component. However, our real-world findings directly counter this concern. In this Medicare population with a mean age of 76 years and a substantial comorbidity burden, VEN-based therapy was not only feasible but associated with markedly superior outcomes. A majority of VEN-treated patients in our cohort received V + O (61%), and these patients demonstrated significantly better OS and TTNT compared with cBTKis and other therapies. Importantly, TTNT benefits remained strong, and OS advantages persisted even when restricting the analysis to V + O recipients. These results demonstrate that VEN-based therapy—including fixed-duration V + O—can be effectively delivered in older, medically complex patients and may offer substantial advantages over continuous cBTKi therapy in frontline CLL treatment.

Overall, our study has several notable strengths, including its large, nationally representative sample of more than 10,000 Medicare beneficiaries and a median follow-up exceeding two years. Nonetheless, some limitations warrant consideration. First, this was a real-world observational study, and although multivariable Cox regression controlled for patient sociodemographic and clinical characteristics, residual confounding from unmeasured characteristics may remain. For example, Medicare claims do not include detailed clinical prognostic information, such as Rai or Binet stage, cytogenetic and molecular markers such as del17p/TP53 mutation or IGHV mutation status, which influence treatment selection and are associated with OS and TTNT in CLL. Additionally, Medicare claims data do not contain immunohistochemistry results or other biomarker assay data, and therefore, BCL2 expression could not be directly assessed in the study. However, BCL2 expression testing is not routinely required for VEN initiation in clinical practice. Likewise, claims data do not contain information on frailty, performance status, or other clinical factors that may affect both the choice of frontline therapy and subsequent outcomes. In addition, observed baseline differences between treatment groups, including age and prior CLL-related hospitalization, suggest the potential for channeling bias in treatment selection. This also introduces the potential for unmeasured confounding. To assess robustness, we conducted an E-value sensitivity analysis; the observed associations between VEN-based regimens and improved outcomes relative to cBTKis would require an unmeasured confounder associated with both treatment choice and OS/TTNT by a hazard ratio of at least 2.32 (OS) and at least 4.82 (TTNT) to fully explain the effects. Although unmeasured confounding cannot be excluded, these findings appear moderately robust but should nevertheless be interpreted with these limitations in mind. Second, we were also unable to directly measure progression-free survival, instead using TTNT as a validated proxy, which may reflect not only disease progression but also treatment intolerance, adverse events, adherence, and clinical decision-making, as claims data do not capture the specific reason for treatment discontinuation or switching. Although the sensitivity analysis, in which intra-class cBTKi switching was not considered a new line of therapy, yielded findings generally consistent with the primary analysis, limitations inherent to claims-based TTNT measures remain and should be considered when interpreting the results. This may be particularly relevant for cBTKi therapies, where treatment discontinuation due to toxicity or intolerance may occur before disease progression, potentially leading TTNT to underestimate true progression-free survival relative to fixed-duration VEN-based regimens. Additionally, the “other regimens” category included heterogeneous treatment approaches, including anti-CD20 monotherapy, chemotherapy, and chemoimmunotherapy, which may differ in patient characteristics, treatment intent, and patterns of clinical use. Although this grouping was intended to reflect major frontline treatment approaches used in real-world care, the heterogeneity of this category should be considered when interpreting comparisons. Third, our findings may not generalize to patients enrolled in Medicare Advantage or to younger, commercially insured populations; however, they are consistent with studies previously published [8]. Finally, given the latest available years of data at the time of our analysis, our study did not have a sizeable number of patients on newer cBTKi agents or combination regimens. Despite these limitations, our analysis of 100% Medicare FFS claims suggests frontline VEN-based therapy can lead to compelling real-world clinical outcomes in older adults with CLL. Our results also underscore the importance of continued real-world evidence generation to inform frontline treatment selection, especially as newer agents and combination regimens (such as VEN plus acalabrutinib, which was only recently recommended in the 2025 NCCN guidelines) are integrated into practice. Future studies with longer follow-up and molecular characterization may be helpful to further personalize frontline therapy selection.

## 5. Conclusions

Among older adults with CLL treated in routine U.S. practice, frontline VEN-based regimens were associated with superior overall survival and time to next treatment compared with cBTKis and other therapies. These findings suggest that VEN-based therapy may offer the most favorable real-world effectiveness among older adults. Given the growing number of treatment options for CLL, our real-world results may inform treatment selection in older adults, where optimizing outcomes and minimizing the need for subsequent treatment are especially beneficial.

## Figures and Tables

**Table 1:** Sample Characteristics.

	VEN-Based Regimens (N = 1503)	cBTKi-Based Regimens (N = 5956)	Other Regimens (N = 3490)
Age, mean (SD)	76.2 (5.8)	78.3 (6.6)	78.3 (6.5)
Age categories, years			
65–69	11.6%	7.2%	6.2%
70–74	32.8%	25.2%	25.9%
75–79	27.9%	27.2%	28.5%
80+	27.7%	40.4%	39.3%
Sex			
Male	64.1%	56.5%	55.3%
Female	35.9%	43.5%	44.7%
Race			
White	92.1%	91.7%	92.4%
Non-white	7.9%	8.3%	7.6%
Black	2.9%	3.9%	3.2%
Hispanic	NR	0.6%	0.5%
Other	NR	3.8%	4.0%
Census Region			
Northeast	16.9%	21.3%	19.2%
Midwest	24.6%	23.8%	24.9%
South	38.9%	37.0%	36.4%
West	19.6%	17.9%	19.5%
Metropolitan Status			
Urban	79.6%	80.0%	81.1%
Rural	20.4%	20.0%	18.9%
Part D Low-Income Subsidy (LIS) and Dual Eligibility status			
Dual LIS	3.5%	6.6%	5.0%
Non-dual LIS	3.1%	4.0%	3.2%
Non-LIS	93.4%	89.4%	91.9%
Part D Drug Benefit Type			
Basic alternative	18.2%	18.4%	19.2%
Enhanced alternative	56.0%	52.5%	56.8%
Other	25.8%	29.1%	23.9%
Social Deprivation Index (SDI) quartiles			
1 (Least disadvantaged)	31.1%	29.8%	31.3%
2 (Slightly disadvantaged)	29.3%	27.7%	27.4%
3 (More disadvantaged)	23.8%	24.1%	22.7%
4 (Most disadvantaged)	15.8%	18.4%	18.5%
Number of Elixhauser comorbidities in the 12-month pre-index period			
0–2	19.0%	17.8%	10.9%
3–4	26.2%	28.1%	22.5%
5–7	32.5%	34.1%	37.4%
8–10	16.8%	14.6%	21.2%
11+	5.4%	5.4%	8.0%
CLL-related hospitalization in the 12-month pre-index period	30.8%	26.3%	35.1%
Frontline CLL treatment initiation year			
2019	13.7%	19.0%	17.8%
2020	28.1%	30.2%	25.8%
2021	28.4%	26.2%	23.8%
2022	29.7%	24.7%	32.6%

Note: NR = Not reported per CMS policy prohibiting display of cell sizes n < 11. Index date = date of index treatment initiation. VEN: venetoclax; cBTKi: Covalent Bruton Tyrosine Kinase Inhibitor; SD: Standard deviation; LIS: Low Income Subsidy; SDI: Social Deprivation Index; CLL: Chronic Lymphocytic Leukemia.

**Table 2:** Cox Regression Results: Overall Survival among Medicare Beneficiaries with Frontline CLL.

Covariates	HR	95% CI		*p*-Value
Index treatment				
VEN	REF			
cBTKi	1.48	1.31	1.67	<0.0001
Other	1.66	1.47	1.89	<0.0001
Age categories, years				
65–69	REF			
70–74	1.06	0.89	1.27	0.50
75–79	1.56	1.31	1.85	<0.0001
80+	2.67	2.25	3.15	<0.0001
Sex				
Male	REF			
Female	0.75	0.70	0.80	<0.0001
Race				
White	REF			
Non-white				
Black	1.07	0.90	1.27	0.44
Hispanic	1.01	0.64	1.59	0.96
Other	0.84	0.68	1.04	0.11
Census Region				
Northeast	REF			
Midwest	1.10	0.99	1.22	0.08
South	1.06	0.96	1.17	0.24
West	1.02	0.91	1.15	0.67
Metropolitan Status				
Urban	REF			
Rural	1.20	1.10	1.30	<0.0001
Part D LIS and Dual Eligible				
Dual LIS	1.17	1.01	1.35	0.03
Non-dual LIS	1.34	1.14	1.58	<0.001
Non-LIS	REF			
Part D Drug Benefit Type				
Enhanced alternative	0.95	0.89	1.02	0.16
Not enhanced	REF			
Social Deprivation Index (SDI) quartiles				
1 (Least disadvantaged)	REF			
2 (Slightly disadvantaged)	0.99	0.90	1.08	0.76
3 (More disadvantaged)	1.00	0.91	1.10	0.96
4 (Most disadvantaged)	1.00	0.90	1.11	0.98
Number of Elixhauser comorbidities in the 12-month pre-index period				
0–2	REF			
3 to 4	1.31	1.14	1.51	<0.001
5 to 7	1.88	1.65	2.14	<0.0001
8 to 10	2.73	2.37	3.15	<0.0001
11+	4.17	3.55	4.90	<0.0001
CLL-related hospitalization in the 12-month pre-index period	1.27	1.18	1.38	<0.0001
Index year of treatment initiation				
2019	1.18	1.08	1.29	<0.001
2020	1.12	1.04	1.22	0.01
2021 or 2022	REF			

HR: Hazard Ratio; CI: Confidence Interval; REF = Reference; VEN: Venetoclax; cBTKi: Covalent Bruton Tyrosine Kinase Inhibitor; LIS: Low Income Subsidy; SDI: Social Deprivation Index; CLL: Chronic Lymphocytic Leukemia.

**Table 3:** Cox Regression Results: Time-to-Next-Treatment among Medicare Beneficiaries with Frontline CLL.

Covariates	HR	95% CI		*p*-Value
Index treatment				
VEN	REF			
cBTKi	2.69	2.23	3.26	<0.0001
Other	6.47	5.32	7.86	<0.0001
Age categories, years				
65–69	REF			
70–74	1.11	0.94	1.32	0.23
75–79	1.19	1.00	1.41	0.05
80+	1.09	0.92	1.30	0.30
Sex				
Male	REF			
Female	1.04	0.96	1.13	0.31
Race				
White	REF			
Non-white				
Black	0.92	0.73	1.16	0.48
Hispanic	1.09	0.59	1.99	0.79
Other	1.12	0.92	1.37	0.27
Census Region				
Northeast	REF			
Midwest	0.91	0.80	1.03	0.15
South	0.95	0.85	1.07	0.42
West	1.07	0.94	1.22	0.31
Metropolitan Status				
Urban	REF			
Rural	1.01	0.91	1.12	0.82
Part D LIS and Dual Eligible				
Dual LIS	0.86	0.70	1.05	0.15
Non-dual LIS	1.24	1.01	1.53	0.04
Non-LIS	REF			
Part D Drug Benefit Type				
Enhanced alternative	0.98	0.90	1.07	0.69
Not enhanced	REF			
Social Deprivation Index (SDI) quartiles				
1 (Least disadvantaged)	REF			
2 (Slightly disadvantaged)	1.00	0.90	1.11	0.98
3 (More disadvantaged)	0.96	0.86	1.08	0.54
4 (Most disadvantaged)	1.00	0.88	1.13	0.99
Number of Elixhauser comorbidities in the 12-month pre-index period				
0–2	REF			
3 to 4	1.00	0.88	1.14	0.99
5 to 7	1.00	0.88	1.13	1.00
8 to 10	0.88	0.75	1.03	0.10
11+	0.68	0.53	0.86	<0.001
CLL-related hospitalization in the 12-month pre-index period	1.18	1.06	1.32	<0.001
Index year of treatment initiation				
2019	1.21	1.09	1.35	<0.001
2020	1.17	1.07	1.29	<0.001
2021 or 2022	REF			

HR: Hazard Ratio; CI: Confidence Interval; REF = Reference; VEN: Venetoclax; cBTKi: Covalent Bruton Tyrosine Kinase Inhibitor; LIS: Low Income Subsidy; SDI: Social Deprivation Index; CLL: Chronic Lymphocytic Leukemia.

## Data Availability

AbbVie is committed to responsible data sharing regarding the clinical trials we sponsor. This includes access to anonymized, individual, and trial-level data (analysis data sets), as well as other information (e.g., protocols, clinical study reports, synopses, or statistical analysis plans), as long as the trials are not part of an ongoing or planned regulatory submission. These clinical trial data can be requested by any qualified researchers who engage in rigorous, independent, scientific research, and will be provided following review and approval of a research proposal, Statistical Analysis Plan (SAP), and execution of a Data Use Agreement (DUA). Data requests can be submitted at any time after approval in the US and Europe and after acceptance of this manuscript for publication. The data will be accessible for 12 months, with possible extensions considered. For more information on the process or to submit a request, visit the following link: https://vivli.org/ourmember/abbvie/ (accessed on 2 June 2026), and then select “Home”.

## Supplementary Materials

The following supporting information can be downloaded at https://www.mdpi.com/article/10.3390/cancers18121902/s1, Supplementary Methods detailing: Approach Identifying Frontline CLL Patients, Classification of Lines of Therapy, Rules For Outcome Definition, Censoring Rules for Outcome Analysis, References for Supplementary Information Provided; Supplementary Table S1: List of CLL Therapies Included in the Analysis; Supplementary Table S2: Summary of Frontline CLL Treatment Regimen, Categories Included in the Analysis; Supplementary Table S3: V + O (n = 910) Sensitivity Analysis: Cox Regression Results: Overall Survival among Medicare Beneficiaries with Frontline CLL; Supplementary Table S4: V + O (n = 910) Sensitivity Analysis: Cox Regression Results: Time-to-Next-Treatment among Medicare Beneficiaries with Frontline CLL; Supplementary Table S5: Intra-class Switching cBTKi Sensitivity Analysis: Cox Regression Results: Time-to-Next-Treatment among Medicare Beneficiaries with Frontline CLL; Supplementary Figure S1: V + O (n = 910) Sensitivity Analysis: Kaplan-Meier Curve of Overall Survival among Medicare Beneficiaries with Frontline CLL; Supplementary Figure S2: V + O (n = 910) Sensitivity Analysis: Kaplan-Meier Curve of Time-to-Next-Treatment among Medicare Beneficiaries with Frontline CLL; Supplementary Figure S3: Intra-class Switching cBTKi Sensitivity Analysis: Kaplan-Meier Curve of Time-to-Next-Treatment among Medicare Beneficiaries with Frontline CLL.

## Abbreviations

1L	First-line
2L	Second-line
BCL-2i	B-cell lymphoma 2 inhibitor
BTK	Bruton’s tyrosine kinase
cBTKi	Covalent Bruton’s tyrosine kinase inhibitor
CCW	Chronic conditions warehouse
CI	Confidence interval
CLL	Chronic lymphocytic leukemia
CMS	Centers for Medicare & Medicaid Services
CT/CIT	Chemotherapy/chemoimmunotherapy
FFS	Fee-for-service
FTD	Fixed-duration treatment
HR	Hazard ratio
IGHV	Immunoglobulin heavy chain variable region
IQR	Interquartile range
LIS	Low-income subsidy
NCCN	National Comprehensive Cancer Network
NR	Not reached
OOP	Out-of-pocket
OS	Overall survival
PFS	Progression-free survival
RWE	Real-world evidence
SD	Standard deviation
SDI	Social Deprivation Index
SLL	Small lymphocytic lymphoma
TTNT	Time to next treatment
VEN	Venetoclax
V + O	Venetoclax plus obinutuzumab
