# Supplementary material for: Real-World Overall Survival and Time to Next Treatment Among Medicare Beneficiaries with Chronic Lymphocytic Leukemia in the Frontline Setting
Source: Cancers (Basel). 2026 Jun 11;18(12):1902. doi: 10.3390/cancers18121902 (PMC13297127; doi:10.3390/cancers18121902)
Supplement: Supplementary file 1 [file cancers-18-01902-s001.zip › cancers-4315279-supplementary.pdf]

SUPPLEMENT

Real-world overall survival and time to next treatment among Medicare beneficiaries with chronic lymphocytic leukemia in the frontline setting

Scott F. Huntington[1], Justin T. Puckett[2], Beenish S. Manzoor[3], Nnadozie Emechebe[3], Sophia S. Li[4], Sachin Kamal-Bahl[2], Carolina Reyes[4], Holly Budlong[3], Jalpa A. Doshi[5]

[1] Yale University, New Haven, CT, USA; [2] COVIA Health Solutions, Lansdale, PA, USA; [3] AbbVie Inc., North Chicago, IL, USA; [4] Genentech Inc., South San Francisco, CA, USA[5] University of Pennsylvania, Philadelphia, PA, USA

SUPPLEMENTARY METHODS

Identifying Front-line CLL Patients

Consistent with prior published approaches for identifying front-line CLL patients in Medicare claims data,[1] beneficiaries were required to meet the following sample selection criteria: (1) continuous fee-for-service Medicare Part A, B, and D coverage for at least 36 months before the index date, (2) continuous fee-for-service Medicare Part A, B, and D coverage for at least 12 months or until death after the index date, (3) ≥1 diagnoses of CLL (C91.1x or C83.0x) on or 12-months before the index date (i.e. pre-index period) AND 12-month after index date (or until death), (4) no evidence of ≥1 diagnosis of other conditions for which cBTKis or venetoclax are indicated (i.e., acute myeloid leukemia [AML], mantle cell lymphoma [MCL], marginal zone lymphoma [MZL], Waldenström macroglobulinemia, or chronic graft versus host disease) during the 12-month pre-index period OR ≥1 diagnoses in the 12-month post-index period or until death, and (5) no evidence of CLL treatment in the 36-month pre-index period (except for anti-CD20 [obinutuzumab, rituximab, biosimilars] in the 4-week pre-index period) to optimize selection of beneficiaries initiating front-line CLL therapy.

Classifying Lines of Therapy

Note: *Lines of therapy refers to the treatment regimen and not a specific drug. A line of therapy is made up of one or more drugs (i.e., a patient can be receiving a single drug or a combination regimen).*

(1) Individual and combination chemotherapies will be identified by Healthcare Common Procedural Coding System codes and National Drug Codes specific to the agents considered (see **Appendix A2** for list of CLL agents included in the study).

(2) To identify a line of therapy, the service date for the **first claim** for any CLL drug therapy occurring within an individual's follow up period after CLL diagnosis (i.e., index date) will be identified.
   a. All CLL therapies filled or administered **during or within 30 days** of the date of the first identified CLL drug fill (or infusion) will be considered part of first-line therapy.
   b. The principle is the same for subsequent lines of therapy – all *other* CLL therapies filled or administered during or within 30 days of the date of the first CLL drug fill (or infusion) identified in that subsequent line of therapy will be considered part of that regimen.

(3) Patients will be assumed to end the previous line of therapy and start a new line of therapy when one of the following scenarios (a) or (b) occur.
   a. Initiation of a new therapy **not part of the most recent prior line of treatment** (i.e., for 2L, you would look to 1L; for 3L, you would look to 2L). This includes two possibilities:
      i. **Switching** medications during follow-up will be considered starting a new line of therapy. ***Note:*** *We will not consider removal of a treatment to be a new line of therapy, as this is expected in real-world clinical practice.*
      ii. **Adding** a medication will be considered starting a new line of therapy. The medication must be added **outside** of the 30-day period used to identify a regimen for the specific line of treatment.
      Special Considerations for anti-CD20s: *Addition of anti-CD20 within 90 days of the index date will not be considered the start of a new line of therapy as this is expected in real-world clinical practice.*
   b. Re-initiation of at least one or all of the agents (with the exception of rituximab) in the regimen belonging to the **most recent prior line of treatment** following a **treatment-free interval of >180 days from the "end date" of the latest drug in that regimen** (i.e., the drug in the regimen whose end date extends the furthest as defined below)
      i. For oral Part D medications (e.g., ibrutinib), the "end date" of treatment will be the date of the **last** prescription fill plus the days' supply of that prescription.
      ii. For IV-administered Part B drugs including rituximab, the "end date" of the treatment will be the date of the **last** Part B drug administration plus 29 days.
   c. In addition to the previous line of therapy ending when the patient meets any of the above two criteria (a) or (b), patient death, entering hospice, or reaching the end of the study period will also be used to classify the end of a line of therapy.
      i. Patients who die during follow up, enter hospice, or reached the end of the study period will be considered censored at the time of those events.

Rules for Outcome Definition

A new therapy introduced more than 30 days after the initial first line (1L) regimen was considered to trigger a new line of treatment, regardless of whether the patient had discontinued their original regimen. The exception to this rule was anti-CD20 agents, which could be added within 90 days of the index date without constituting a new line, reflecting real-world clinical practice. Discontinuation of a regimen was defined as a continuous 90-day gap in therapy, based on prior published methodology.[2] If a patient discontinued a 1L regimen and then restarted the same regimen, this only counted as a new line if the re-initiation occurred more than 180 days after the end date of the last drug in the regimen. For the primary analysis, switching within the same drug class (e.g., from ibrutinib to acalabrutinib) was regarded as initiating a new line of therapy.

Censoring Rules for Outcome Analysis

For overall survival (OS), death was considered the event and patients were censored at end of follow-up. For time to next treatment (TTNT), initiation of a subsequent therapy was considered an event, while patients were censored at death, hospice entry, enrollment into Medicare Advantage, or the end of follow-up.

Supplementary References

1. National Comprehensive Cancer Network. Treatment by Cancer Type. NCCN Clinical Practice Guidelines in Oncology Version 3.2025 - CLL/SLL. April 2, 2025. Accessed July 11, 2025. https://www.nccn.org/professionals/physician_gls/pdf/cll.pdf
2. Huntington SF, Rhodes JM, Manzoor BS, et al. Real-World Treatment Patterns After Discontinuation of Venetoclax or BTKis in the Frontline Setting Among Older Adults With Chronic Lymphocytic Leukemia. *JCO Oncol Pract*. 2025;21(8):1124-1133. doi:10.1200/OP.24.00220

SUPPLEMENTARY TABLES

Supplementary Table S1. List of CLL Therapies Included in the Analysis

Generic Name	Class
venetoclax	BCL-2i
acalabrutinib	cBTKi
ibrutinib	cBTKi
zanubrutinib	cBTKi
obinutuzumab	aCD20
ofatumumab	aCD20
rituximab and hyaluronidase	aCD20
rituximab	aCD20
rituximab-abbs	aCD20
rituximab-arrx	aCD20
rituximab-pvvr	aCD20
alemtuzumab	aCD52
bendamustine	Chemotherapy
bendamustine hcl	Chemotherapy
chlorambucil	Chemotherapy
cladribine	Chemotherapy
cyclophosphamide	Chemotherapy
doxorubicin	Chemotherapy
etoposide	Chemotherapy
fludarabine	Chemotherapy
mitoxantrone	Chemotherapy
pentostatin	Chemotherapy
vincristine	Chemotherapy
vinorelbine	Chemotherapy
lenalidomide	Immunomodulator
lisocabtagene maraleucel	CAR-T
pirtobrutinib	ncBTKi
copanlisib	PI3Ki
duvelisib	PI3Ki
idelalisib	PI3Ki
umbralisib	PI3Ki

CLL: Chronic Lymphocytic Leukemia; BCL-2i: BCL-2 inhibitor; cBTKi: Covalent Bruton Tyrosine Kinase Inhibitor; aCD20: Anti-CD20 antibody; aCD52: Anti-CD52 antibody; CAR-T: Chimeric Antigen Receptor T-cell therapy; ncBTKi: Non-covalent Bruton Tyrosine Kinase Inhibitor; PI3Ki: Phosphoinositide 3-kinase inhibitor

Supplementary Table S2. Summary of Frontline CLL Treatment Regimen Categories Included in the Analysis

Regimen category	Representative agents included in analysis	Drug class	Primary molecular target	Treatment nature
VEN-based regimens	Venetoclax + obinutuzumab; venetoclax monotherapy; venetoclax + acalabrutinib; venetoclax + zanubrutinib	BCL-2 inhibitor	BCL-2	Primarily fixed-duration
cBTKi-based regimens	Ibrutinib; acalabrutinib; zanubrutinib; cBTKi + anti-CD20; cBTKi + other agents	Covalent BTK inhibitor	BTK	Continuous until progression, intolerance, or discontinuation
Other regimens	Anti-CD20 monoclonal antibody: obinutuzumab; ofatumumab; rituximab and hyaluronidase; rituximab; rituximab-abbs; rituximab-arrx; rituximab-pvvr.	Anti-CD20 monoclonal antibody and/or chemotherapy/chemoimmunotherapy	CD20 and/or DNA/cell division	Variable; generally non-targeted or finite-duration frontline regimens
	Single-agent chemotherapy: chlorambucil; bendamustine; bendamustine hcl; fludarabine; cyclophosphamide; cladribine; doxorubicin; etoposide; mitoxantrone; pentostatin; vincristine; vinorelbine. Chemoimmunotherapy: bendamustine + rituximab; chlorambucil + obinutuzumab; fludarabine + rituximab; fludarabine + cyclophosphamide + rituximab; cyclophosphamide + rituximab; chlorambucil + rituximab; bendamustine + obinutuzumab.			

CLL, chronic lymphocytic leukemia; VEN, venetoclax; cBTKi, covalent Bruton's tyrosine kinase inhibitor; BTK, Bruton's tyrosine kinase; BCL-2, B-cell lymphoma 2. The "other regimens" category includes both anti-CD20 monotherapy and chemotherapy/chemoimmunotherapy regimens commonly used in frontline practice during the study period.

Supplementary Table S3. V+O (n=910) Sensitivity Analysis: Cox Regression Results: Overall Survival among Medicare Beneficiaries with Front-line CLL

Covariates	HR	95% CI		p-value
Index treatment				
V+O	**REF**			
cBTKi	1.61	1.37	1.89	<.0001
Other	1.81	1.53	2.13	<.0001
Age categories, years				
65-69	**REF**			
70-74	1.06	0.88	1.28	0.54
75-79	1.58	1.31	1.89	<.0001
80+	2.74	2.30	3.27	<.0001
Sex				
Male	**REF**			
Female	0.76	0.70	0.81	<.0001
Race				
White	**REF**			
Non-white				
Black	1.10	0.92	1.31	0.30
Hispanic	0.98	0.62	1.55	0.93
Other	0.84	0.68	1.05	0.13
Census Region				
Northeast	**REF**			
Midwest	1.09	0.98	1.21	0.13
South	1.05	0.95	1.16	0.33
West	1.03	0.91	1.15	0.65
Metropolitan Status				
Urban	**REF**			
Rural	1.19	1.09	1.30	<.0001
Part D LIS and Dual Eligible				
Dual LIS	1.16	1.00	1.34	0.05
Non-dual LIS	1.38	1.17	1.62	0.00
Non-LIS	**REF**			
Part D Drug Benefit Type				
Enhanced alternative	0.94	0.88	1.01	0.12
Not enhanced	**REF**			
Social Deprivation Index (SDI) quartiles				
1 (Least disadvantaged)	**REF**			
2 (Slightly disadvantaged)	0.98	0.89	1.08	0.69
3 (More disadvantaged)	1.00	0.91	1.11	0.94
4 (Most disadvantaged)	0.99	0.89	1.10	0.81
Number of Elixhauser comorbidities in the 12-month pre-index period				
0-2	**REF**			

3 to 4	1.30	1.13	1.50	0.00
5 to 7	1.87	1.63	2.14	<.0001
8 to 10	2.77	2.40	3.20	<.0001
11+	4.19	3.55	4.94	<.0001
CLL-related hospitalization in the 12-month pre-index period	1.25	1.16	1.36	<.0001
Index year of treatment initiation				
2019	1.19	1.08	1.30	0.00
2020	1.13	1.04	1.23	0.00
2021 or 2022	**REF**			

CLL: Chronic Lymphocytic Leukemia; V+O, venetoclax plus obinutuzumab; cBTKi, covalent Bruton Tyrosine Kinase inhibitor; CLL, chronic lymphocytic leukemia; LIS, Limited Income Subsidy; SDI, Social Deprivation Index; HR, hazard ratio; CI, confidence interval; REF, reference category.

Supplementary Table S4. V+O (n=910) Sensitivity Analysis: Cox Regression Results: Time-to-Next-Treatment among Medicare Beneficiaries with Front-line CLL

Covariates	HR	95% CI		p-value
Index treatment				
V+O	**REF**			
cBTKi	3.55	2.69	4.68	<.0001
Other	8.56	6.47	11.31	<.0001
Age categories, years				
65-69	**REF**			
70-74	1.12	0.94	1.33	0.22
75-79	1.20	1.00	1.43	0.05
80+	1.10	0.92	1.31	0.28
Sex				
Male	**REF**			
Female	1.03	0.95	1.12	0.43
Race				
White	**REF**			
Non-white				
Black	0.90	0.71	1.14	0.39
Hispanic	1.12	0.61	2.05	0.71
Other	1.10	0.89	1.35	0.38
Census Region				
Northeast	**REF**			
Midwest	0.92	0.81	1.05	0.20
South	0.97	0.86	1.09	0.58
West	1.08	0.94	1.23	0.28
Metropolitan Status				
Urban	**REF**			
Rural	1.01	0.91	1.12	0.88
Part D LIS and Dual Eligible				
Dual LIS	0.84	0.68	1.03	0.10
Non-dual LIS	1.23	0.99	1.52	0.06
Non-LIS	**REF**			
Part D Drug Benefit Type				
Enhanced alternative	0.97	0.89	1.05	0.43
Not enhanced	**REF**			
Social Deprivation Index (SDI) quartiles				
1 (Least disadvantaged)	**REF**			
2 (Slightly disadvantaged)	1.00	0.89	1.11	0.94
3 (More disadvantaged)	0.96	0.85	1.08	0.49
4 (Most disadvantaged)	1.00	0.88	1.14	0.97
Number of Elixhauser comorbidities in the 12-month pre-index period				
0-2	**REF**			

3 to 4	1.01	0.88	1.15	0.92
5 to 7	1.01	0.89	1.14	0.91
8 to 10	0.86	0.73	1.01	0.06
11+	0.67	0.52	0.85	0.00
CLL-related hospitalization in the 12-month pre-index period	1.20	1.07	1.33	0.00
Index year of treatment initiation				
2019	1.19	1.06	1.32	0.00
2020	1.16	1.05	1.27	0.00
2021 or 2022	**REF**			

CLL: Chronic Lymphocytic Leukemia; V+O, Venetoclax plus obinutuzumab; cBTKi, covalent Bruton Tyrosine Kinase inhibitor; CLL, chronic lymphocytic leukemia; LIS, Limited Income Subsidy; SDI, Social Deprivation Index; HR, hazard ratio; CI, confidence interval; REF, reference category.

Supplementary Table S5. Intra-class Switching cBTKi Sensitivity Analysis: Cox Regression Results: Time-to-Next-Treatment among Medicare Beneficiaries with Front-line CLL

Covariates	HR	95% CI		p-value
Index treatment				
VEN	**REF**			
cBTKi	2.04	1.68	2.48	<.0001
Other	6.52	5.35	7.95	<.0001
Age categories, years				
65-69	**REF**			
70-74	1.09	0.91	1.32	0.35
75-79	1.20	1.00	1.45	0.05
80+	1.05	0.88	1.26	0.59
Sex				
Male	**REF**			
Female	1.04	0.95	1.14	0.39
Race				
White	**REF**			
Non-white				
Black	0.96	0.75	1.23	0.76
Hispanic	1.22	0.64	2.30	0.54
Other	1.06	0.85	1.32	0.62
Census Region				
Northeast	**REF**			
Midwest	0.89	0.77	1.02	0.09
South	0.94	0.83	1.06	0.31
West	1.02	0.88	1.17	0.80
Metropolitan Status				
Urban	**REF**			
Rural	0.96	0.86	1.08	0.50
Part D LIS and Dual Eligible				
Dual LIS	0.81	0.65	1.02	0.07
Non-dual LIS	1.28	1.03	1.60	0.03
Non-LIS	**REF**			
Part D Drug Benefit Type				
Enhanced alternative	1.00	0.91	1.10	0.96
Not enhanced	**REF**			
Social Deprivation Index (SDI) quartiles				
1 (Least disadvantaged)	**REF**			
2 (Slightly disadvantaged)	0.95	0.84	1.06	0.36
3 (More disadvantaged)	0.99	0.88	1.13	0.93
4 (Most disadvantaged)	1.03	0.90	1.18	0.66
Number of Elixhauser comorbidities in the 12-month pre-index period				
0-2	**REF**			

3 to 4	1.02	0.89	1.18	0.76
5 to 7	1.00	0.87	1.14	0.96
8 to 10	0.87	0.74	1.03	0.11
11+	0.70	0.54	0.89	0.00
CLL-related hospitalization in the 12-month pre-index period	1.22	1.09	1.37	0.00
Index year of treatment initiation				
2019	1.29	1.14	1.44	<.0001
2020	1.22	1.10	1.35	0.00
2021 or 2022	**REF**			

CLL: Chronic Lymphocytic Leukemia; V+O, venetoclax plus obinutuzumab; cBTKi, covalent Bruton Tyrosine Kinase inhibitor; CLL, chronic lymphocytic leukemia; LIS, Limited Income Subsidy; SDI, Social Deprivation Index; HR, hazard ratio; CI, confidence interval; REF, reference category.

SUPPLEMENTARY FIGURES

Supplementary Figure S1. V+O (n=910) Sensitivity Analysis: Kaplan-Meier Curve of Overall Survival among Medicare Beneficiaries with Front-line CLL

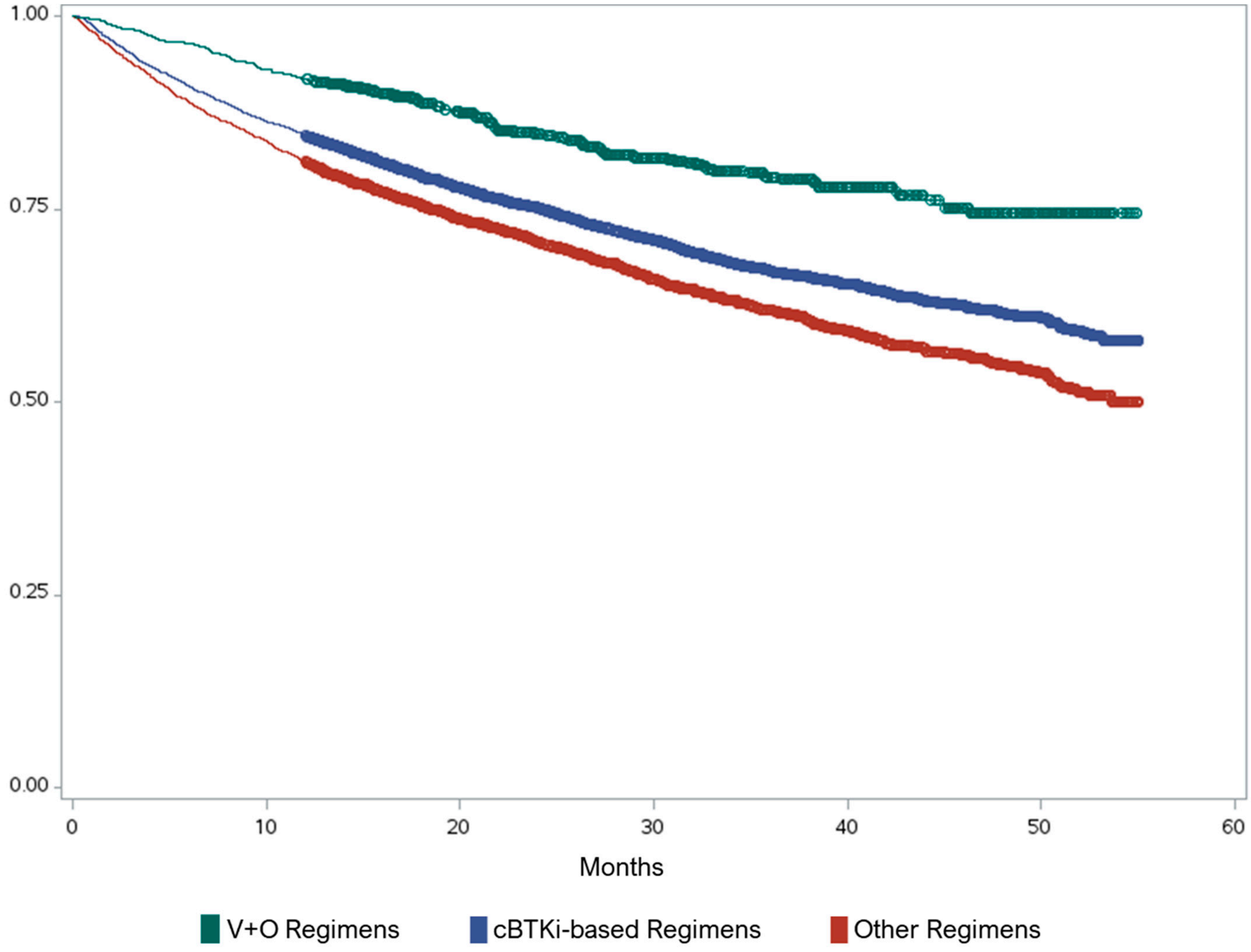

Survival Rates	V+O Regimen	cBTKi-based Regimens	Other Regimens
1-year	92%	85%	81%
2-year	85%	75%	71%
3-year	79%	67%	62%

CLL: Chronic Lymphocytic Leukemia; V+O, venetoclax plus obinutuzumab; cBTKi, covalent Bruton Tyrosine Kinase inhibitor; CLL, chronic lymphocytic leukemia.

Supplementary Figure S2. V+O (n=910) Sensitivity Analysis: Kaplan-Meier Curve of Time-to-Next-Treatment among Medicare Beneficiaries with Front-line CLL

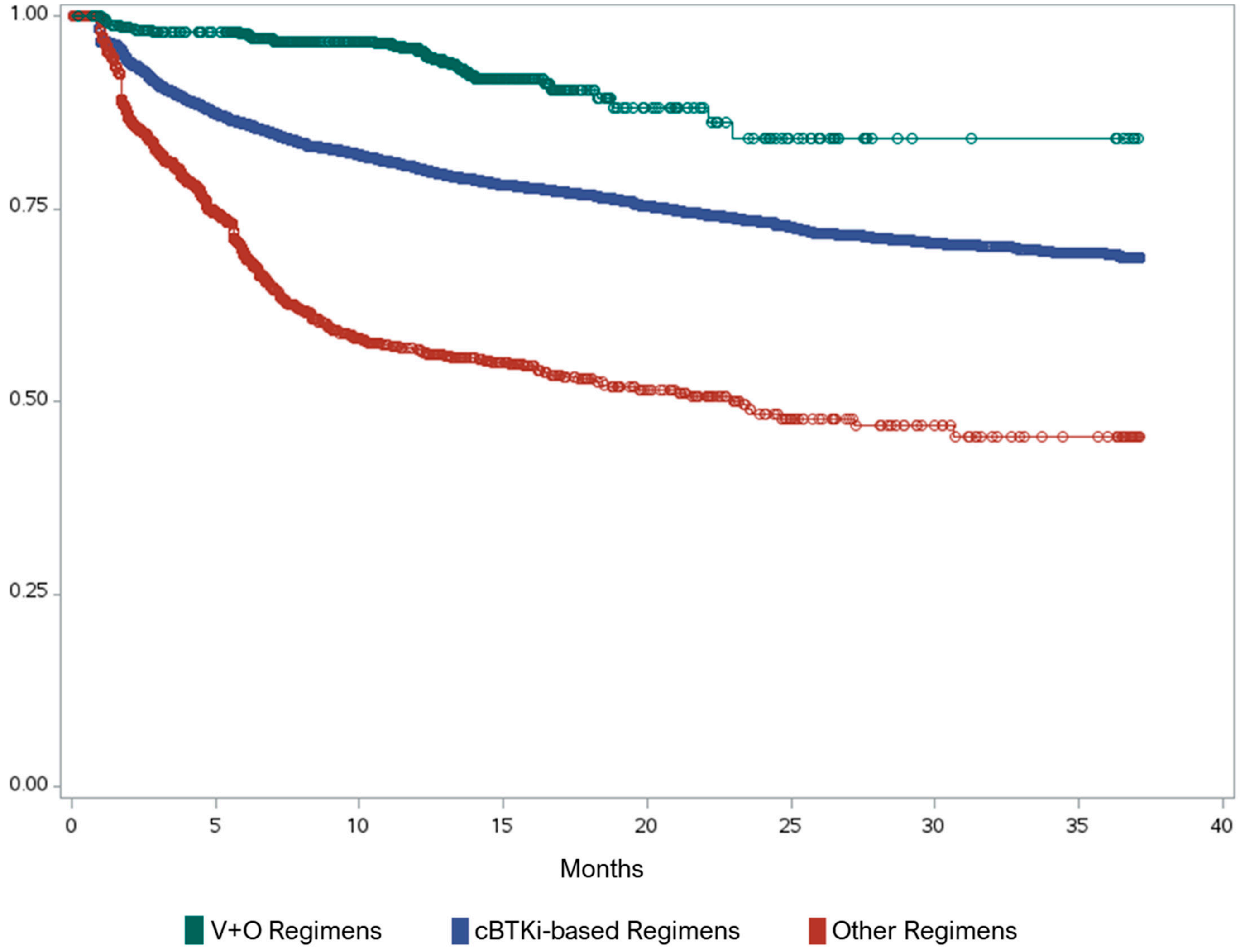

TTNT Rates	V+O Regimens	cBTKi-based Regimens	Other Regimens
1-year	95%	80%	62%
2-year	88%	73%	55%
3-year	88%	69%	52%

CLL: Chronic Lymphocytic Leukemia; V+O, venetoclax plus obinutuzumab; cBTKi, covalent Bruton Tyrosine Kinase inhibitor; CLL, chronic lymphocytic leukemia.

Supplementary Figure S3. Intra-class Switching cBTKi Sensitivity Analysis: Kaplan-Meier Curve of Time-to-Next-Treatment among Medicare Beneficiaries with Front-line CLL

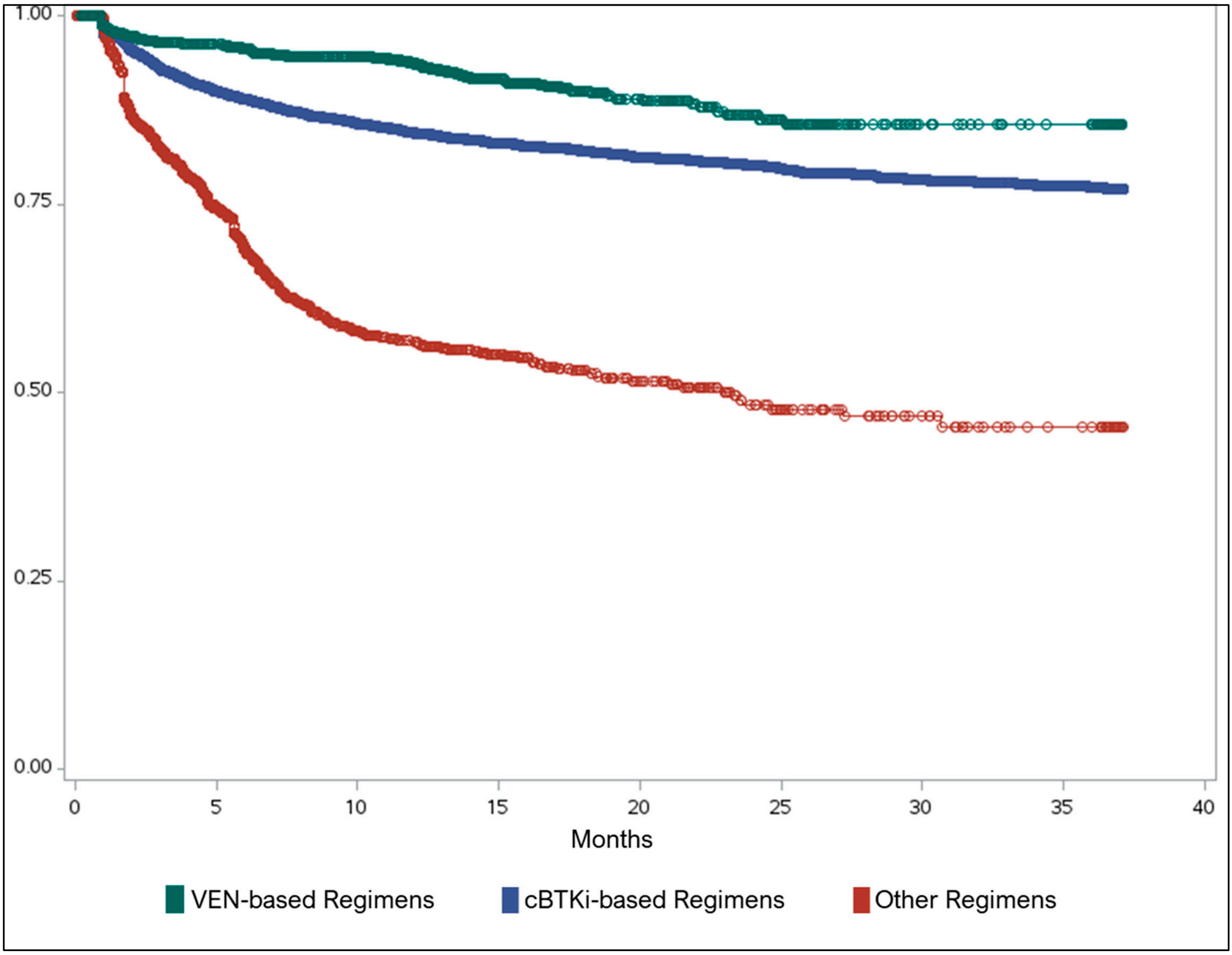

TTNT Rates	VEN-based Regimens	cBTKi-based Regimens	Other Regimens
1-year	93%	84%	62%
2-year	87%	80%	55%
3-year	86%	77%	52%

CLL: Chronic Lymphocytic Leukemia; V+O, venetoclax plus obinutuzumab; cBTKi, covalent Bruton Tyrosine Kinase inhibitor; CLL, chronic lymphocytic leukemia.
